# Prostate motion in magnetic resonance imaging-guided radiotherapy and its impact on margins

**DOI:** 10.1007/s00066-024-02346-z

**Published:** 2025-01-14

**Authors:** Johannes Kusters, René Monshouwer, Peter Koopmans, Markus Wendling, Ellen Brunenberg, Linda Kerkmeijer, Erik van der Bijl

**Affiliations:** Department of Radiation Oncology, Radboud university medical center, Nijmegen, The Netherlands

**Keywords:** Treatment strategy, Margin reduction, Online adaptive radiotherapy, Prostate cancer, MR-Linac

## Abstract

**Purpose:**

This study focused on reducing the margin for prostate cancer treatment using magnetic resonance imaging-guided radiotherapy by investigating the intrafractional motion of the prostate and different motion-mitigation strategies.

**Methods:**

We retrospectively analyzed intrafractional prostate motion in 77 patients with low- to intermediate-risk prostate cancer treated with five fractions of 7.25 Gy on a 1.5 T magnetic resonance linear accelerator. Systematic drift motion was observed and described by an intrafractional motion model. The planning target volume (PTV) margin was calculated in a cohort of 77 patients and prospectively evaluated for geometric coverage in a separate cohort of 24 patients.

**Results:**

The intrafractional model showed that the prostate position starts out of equilibrium for the anterior–posterior (−1.8 ± 3.1 mm) and superior–inferior (1.7 ± 2.6 mm) directions, with relaxation times of 12 and 15 min, respectively. Position verification scans are acquired at 30 min on average. At that time, the transient drift motion becomes indistinguishable from the residual random intrafractional motion. PTV margins can be reduced to 1.8 mm (left–right), 3.2 mm (anterior–posterior), and 2.9 mm (superior–inferior). Evaluation of the overlap with the clinical target volume (CTV) was performed for a total of 120 fractions of 24 patients. The overlap range between the CTV and the PTV was 93–100% and the applied 3‑mm PTV margin for the CTV had a 99.5% averaged geometric overlap for all patients.

**Conclusion:**

A PTV margin reduction to 3 mm is feasible. A patient-specific approach could reduce the margins further.

**Supplementary Information:**

The online version of this article (10.1007/s00066-024-02346-z) contains supplementary material, which is available to authorized users.

## Introduction

The clinical availability of magnetic resonance imaging (MRI)-guided radiotherapy (MRgRT) devices [1, 2] couples the superior soft tissue contrast of MRI to daily plan adaptation. At each fraction, the target and surrounding organs at risk are re-delineated on the MR image to capture changes in anatomy. This online adaptive MRgRT process allows for improved target coverage with low normal tissue toxicity [3, 4], margin reduction [5], and opportunities for isotoxic dose escalation [6–9].

For example, the phase III MIRAGE trial compared adaptive stereotactic MRgRT for prostate cancer (PCa) with reduced margins to standard computed tomography (CT)-based treatments [5] and reported a significantly lower incidence of acute grade 2+ genitourinary (24.4% vs. 43.4%) and gastrointestinal (0.0% vs. 10.5%) toxicities for MRgRT versus CT guidance, respectively.

As a consequence of the online adaptive workflow, treatment sessions using MRgRT take longer than conventional treatments for PCa. In our institution, patient on-couch time was 50 min on average (with an interquartile range of 43–55 min), making intrafractional prostate motion relevant. Intrafractional motion of the prostate has been widely studied using ultrasound [10, 11], cone-beam CT [12, 13], and repeated 2D or 3D MRI [14–22]. The latter used 3D high-speed magnetic resonance imaging sequences to capture motion (3D-cine MRI) [18, 19], correlation with dose delivery based on 2D-cine [20] and 3D-cine MRI [21], and gating [22]. All studies observed random patterns of motion, with an increasing probability of a shifted prostate from the initial position over time.

This study focused on improving MRgRT in PCa patients by determining optimal margins when a threshold is used to determine whether to start a second adaptation to address intrafractional motion during the first plan adaptation.

To this end, we investigate prostate motion during MRgRT fractions, and an intrafractional motion model of the prostate is proposed to interpret the success of this approach. In this model, random forces act together with a restoring force. As an initial condition, a population-averaged out-of-equilibrium position is fitted to our data. As such, we investigate intrafractional prostate motion over the course of approximately an hour and introduce a model that captures the systematic motion of the prostate in combination with random fluctuations. This model helps to find strategies to further fine-tune the online adaptive workflow at MRI linear accelerators (MRI-Linacs), such as reduced or personalized margins [23–25], a second plan adaptation before the start of treatment [19, 26], or gating [22].

## Materials and methods

**Fig. 1:**
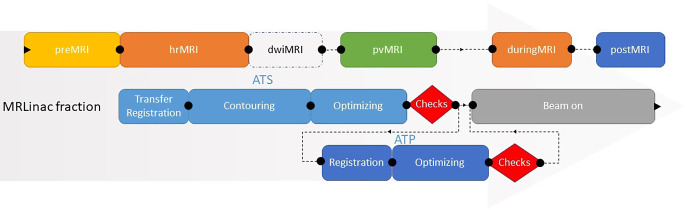
Workflow diagram showing the clinical online ATS-ATP (adapt to shape - adapt to position) processes for treatment generation and delivery. During the ATS workflow, online contouring and plan optimization are performed. After dose calculation of the plan has almost finished, a position-verification scan is acquired. If shifts greater than 3 mm have occurred, a virtual couch shift is applied (ATP workflow). The analysis of prostate motion in this study was based on anatomical imaging of the pre-, higher-resolution (*hr*), position-verification (*pv*), during-, and post-MRI scans taken over the whole fraction when the patient was in the treatment position. Diffusion weighted imaging (dwi) MR images were obtained during delineation of the targets and organs at risk

We studied patients treated for low- and intermediate-risk PCa with five fractions of 7.25 Gy using MRgRT (Unity, Elekta AB, Stockholm, Sweden). All treatments were based on full re-delineation on the MRI of the day (preMRI) followed by full plan optimization; this workflow is referred to as the adapt-to-shape (ATS) workflow [27]. In this time-consuming workflow, the prostate and organs at risk (OARs) are propagated to the preMRI from the reference MRI. The OARs are delineated only within the first centimeter surrounding the planning target volume (PTV). Directly before the start of irradiation, a position-verification MRI is acquired. When a shift of the prostate greater than 3 mm is observed, an additional rigid adaptation (called the adapt-to-position [ATP] workflow) is applied to the ATS plan to correct for translations of the target. A schematic overview of the MRgRT workflow for PCa treatment is shown in Fig. 1.

### Patient cohort

We retrospectively analyzed prostate motion in 77 PCa patients treated between November 2020 and July 2022 with isotropic 5‑mm prostate PTV margins and of 24 patients treated between August 2022 and April 2023 with 3‑mm PTV margins. For intermediate-risk patients, a separate clinical target volume (CTV) for the base of the seminal vesicles was used clinically but was not included in this study. All patients were instructed to empty their bladder 30 min before scanning and treatment. All patients signed informed consent within the registration study Momentum (NCT04075305) [28].

### Imaging

At least five 3D T2-weighted (T2W) MRI scans were acquired each fraction: in addition to the aforementioned first for plan adaptation (pre) and second for position verification (pv) scans, scans were also made approximately halfway through treatment (during) and at the end of irradiation (post). During the plan adaptation phase, a higher-resolution (hr) scan was acquired for additional research purposes. Supplement A gives more details of the obtained MR sequences.

### Quantification of intrafractional prostate motion and margins

The intrafractional motion of the prostate was obtained by determining the translation in the center-of-mass (COM) position. For 77 PCa patients, each fraction was analyzed by applying deformable image registration (Plastimatch v1.9.3 [https://plastimatch.org],  mainly developed by Harvard University, Boston) of the MRI scans of a fraction to the preMRI for the region around the prostate (see Supplement B for more details on the deformable registration).

The differences in extracted COM positions were used to calculate the population systematic mean (µ) and the population systematic (Σ) and population random (σ) residual setup errors. In addition, we calculated the intrafractional random error (σ_intra_).

The values of µ and Σ were estimated by taking the average and standard deviation, respectively, of the per-patient average COM displacement between the average position during irradiation and the scan used for planning, which could be pvMRI (ATS-ATP workflow) or preMRI (ATS workflow). The random errors (σ) were obtained by taking the population mean of the standard deviations of the setup errors. The intrafractional motion *σ*_*intra* _  is estimated from the population mean of the per-fraction standard deviation in position in the pv-, during-, and postMRI.

Prostate PTV margins were calculated considering the results of this study for intrafractional motion and other uncertainties in the accuracy of the MV Linac beam, delineations of contours, penumbras, MV-MR isocenters, and rotations [23, 29]. The benefit of the ATS-ATP workflow over the ATS-only workflow was further demonstrated by simulating the resulting motion of the prostate CTV for different ATP thresholds. The ATP procedure does not allow adaptation to rotations of the target volume, other than by minimizing its effect by a suitable translation. A rotation of the prostate around the apex leads to an superior–inferior (SI) and anterior-posterior (AP) shift of the COM, combined with a residual rotation around the COM. To estimate the effect of residual intrafractional rotations, we also calculated the residual rotation after rigid registration using the COM.

### Prostate gland intrafractional motion model

The distribution of intrafractional motion of the prostate is modelled as the overdamped motion of a particle in a medium on which random forces act, caused, for example, by peristalsis. In such a model, the phenomenological description of the motion of the prostate will be a random walk with a linearly increasing variance of the observed positions with time [11, 30]. When, in addition to the random forces, a harmonic restoring force toward a per-patient equilibrium position of the prostate is added, the variance of the observed positions becomes constant in time, and the observed positions can be described by a Gaussian distribution around the equilibrium position, such as the assumption in classical margin models for intrafractional motion [23]. The width of the distribution depends on the balance between the random and restoring forces. With this model, we added, as a boundary condition, that the prostate begins its intrafractional motion, on average, out of equilibrium. This model features a transient systematic motion toward equilibrium in superposition with random motions. The distribution of observed prostate positions is then described by a generalized Gaussian distribution with a time-dependent mean position $\mathrm{X}\left(t\right)=x_{0}\exp \left[-t/\tau \right]$ and width $\sigma \left(t\right)=\sigma _{i}\sqrt{1+\left(\frac{{\sigma }_{0}^{2}}{{\sigma }_{i}^{2}}-1\right)\exp \left[-2t/\tau \right]}$. In these equations, the population-averaged mean position X(*t*) depends on the observed average out-of-equilibrium prostate position *x*_0_ at t = 0 and the relaxation time *τ*. The time-dependent width evolves from the initial (t = 0) standard deviation of the out-of-equilibrium positions *σ*_0_ toward the static *σ*_*i*_ that describes the intrafractional motion around the equilibrium positions at longer timescales from the moment of laying down. The equations for the average and squared average position were fitted simultaneously to the data for the three main directions using the four parameters *x*_0_, *σ*_0_, *σ*_*i*_, and *τ*. See Supplement C for more details.

### Prospective evaluation of the clinically implemented 3-mm CTV–PTV margin for the prostate gland

Prospective evaluation of the reduced 3‑mm PTV margin, with a 2-mm ATP threshold, was performed for 24 PCa patients. The daily adapted CTV was deformably propagated from the online plan image (pre) to all subsequent MRI-scans. The volumetric overlap of the PTV from the preMRI (in the case of ATS) or pvMRI (in the case of ATS-ATP) with the CTV on the MR scan during treatment was calculated. The margin was considered acceptable if at least 98% of the CTV obtained during the scan overlapped with the PTV for 90% of the patients. We assumed that the PTV was completely covered with 95% of the prescribed dose.

## Results

### Intrafractional motion of COMs and PTV margins

In the ATS-only workflow, an absolute population mean for systematic shift (µ $\cong$ 2 mm) in the AP and SI directions was observed, with a standard deviation of approximately Σ$\cong$ 2.3 mm (Table 1). This finding is in line with the best-fit results of the next paragraph. Motion in the LR direction was very limited and stable (maximum 1 mm). Thus, in an ATS-only workflow, margins larger than 5 mm would be advisable. However, since translations above the threshold triggered the ATS-ATP workflow, a clinical margin reduction was possible. The ATS-ATP workflow with an ATP threshold of 3 mm was successful in eliminating the systematic shift (µ = 0.4 mm) at the cost of a more involved workflow. ATP was performed in 41% of the fractions and led to a smaller residual motion (Σ$\cong$ 1.1 mm, AP, SI). The random motions over the course of treatment were not affected by the workflow, resulting in *σ*_*intra* _  $\cong$ 0.6 mm. Intrafractional rotations of the prostate during the workflow are partially taken into account by translations in COMs. Results for the rotational mean ± SD were −0.1 ± 0.4, 0.0 ± 1.5, and 0.1 ± 0.5 degrees in roll, pitch, and yaw, respectively.

**Table 1:** Motion statistics—systematic mean (µ), systematic deviation (Σ), and random errors (σ + σ_intra_)—for COM (center of mass) translations of the CTV for the ATS (adapt-to-shape) and ATS-ATP (adapt to shape - adapt to position) workflows with different ATP (adapt-to-position) threshold values

	ATS-only workflow			ATP-ATS workflow														
Threshold value (mm)	*–*			0			1			2			3			5		
–	*LR*	*AP*	*SI*	*LR*	*AP*	*SI*	*LR*	*AP*	*SI*	*LR*	*AP*	*SI*	*LR*	*AP*	*SI*	*LR*	*AP*	*SI*
µ (mm)	0.0	1.8	−1.9	0.1	−0.2	0.1	0.1	−0.2	0.1	0.1	0.0	−0.1	0.1	0.4	−0.4	0.0	1.1	−1.2
Σ (mm)	0.9	2.2	2.3	0.5	0.9	0.9	0.5	0.9	0.9	0.5	1.0	0.9	0.7	1.2	1.0	0.8	1.5	1.4
σ (mm)	1.0	1.5	1.5	0.6	1.3	1.2	0.6	1.3	1.2	0.7	1.4	1.3	0.8	1.6	1.5	0.9	1.7	1.7
Σ_intra_ (mm)	0.3	0.6	0.6	0.3	0.6	0.6	0.3	0.6	0.6	0.3	0.6	0.6	0.3	0.6	0.6	0.3	0.6	0.6
*–*				ATP: 100%			ATP: 87%			ATP: 60%			ATP: 41%			ATP: 15%		

All values are in millimeters. The bottom row gives the percentage of fractions where ATP should have been performed given the threshold on the length of the COM displacement vector

The resulting PTV margins for the ATS-ATP workflow using a 2-mm ATP-threshold (Table 2) were reduced to 1.4 mm (LR), 3.1 mm (AP), and 2.9 mm (SI).

**Table 2:** Calculated PTV margins for the ATS and ATS-ATP workflows using a 2-mm ATP threshold

	ATS			ATS-ATP		
mm	LR (mm)	AP	SI	LR	AP	SI
µ (population mean)	0.0	1.8	−1.9	0.1	0.0	−0.1
Σ	0.9	2.2	2.3	0.4	0.9	0.8
Σ_machine	0.2	0.2	0.2	0.2	0.2	0.2
Σ_rotations	0.2	0.5	0.5	0.2	0.5	0.5
σ	1.0	1.5	1.5	0.7	1.4	1.3
σ_intra	0.3	0.6	0.6	0.3	0.6	0.6
σ_penumbra	4.4	4.4	4.4	4.4	4.4	4.4
σ_machine	0.2	0.2	0.2	0.2	0.2	0.2
σ_delineation	0.5	0.5	0.5	0.5	0.5	0.5
σ_rotations(deg)	0.3	0.5	0.5	0.3	0.5	0.5
Margin	2.6	6.2	6.5	1.4	3.1	2.9

### Intrafractional motion model

**Fig. 2:**
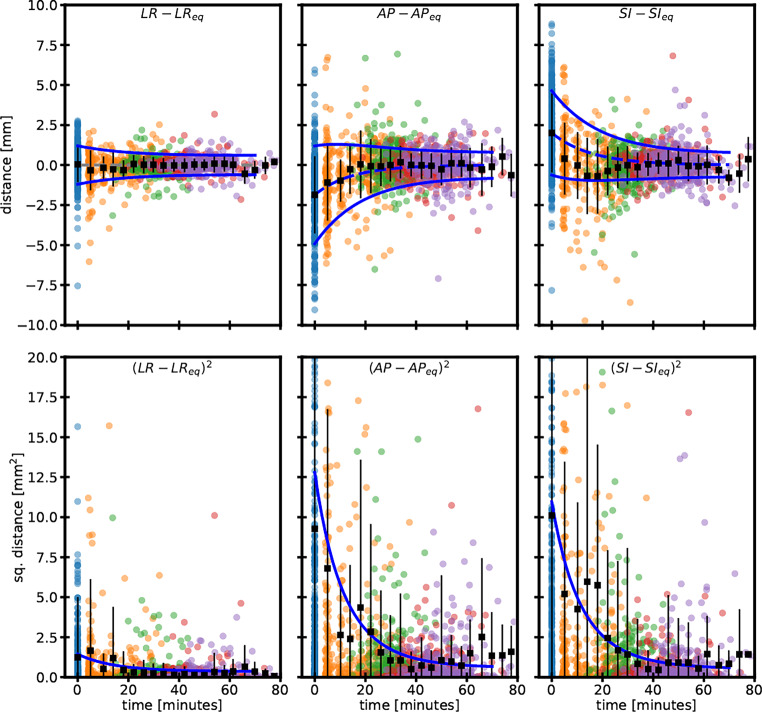
All COM positions of the 77 patients were color coded for the number of the MRI within the fraction, with timing relative to the preMRI. The blue dots are the COMs of the preMRI scans, the orange dots are the COMs of the hrMRI, the green dots are the COMs of the pvMRI, the pink dots are the COMs of the duringMRI scans, and the purple dots are the COMs of the postMRI scans along the three directions: left–right (*LR*), anterior–posterior (*AP*), and superior–inferior (*SI*). The black squares and vertical lines indicate the means and standard deviations, respectively, and are binned in 3‑min time intervals. The blue lines represent the best fits to the data as described by the model in the main text. The solid lines indicate the fitted time-dependent standard deviation around the average position (dashed line)

Fig. 2 depicts all COM positions relative to the inferred equilibrium positions for the three directions LR, AP, and SI, with the blue lines corresponding to the best-fit intrafractional motion model for the average displacement and spread as given in the “Materials and methods” section. The best-fit parameters are shown in Table 3. From the data it is clear that a population-averaged out-of-equilibrium starting position $x_{0}\pm \sigma _{0}$ was present for the AP (−1.8 ± 3.1 mm) and SI (1.7 ± 2.6 mm) directions. In the LR direction, the initial position was fitted to be 0.1 ± 1.1 mm. Fitted relaxation times of 12 and 15 min for the AP and SI directions, respectively, were found. For the LR axis the fit was not stable for the relaxation time and was fixed to be 12 and 15 min, with no change in the fitted results for the other parameters. Since position-verification scans are acquired at 30 min on average, the transient drift motion becomes indistinguishable from the residual random intrafractional motion, explaining the success of the ATS-ATP workflow. Quantitatively, the model is related to the average observed setup uncertainty of the previous section via $\mu _{ATS-Only}\cong X\left(t=0\right)=x_{0}$ and $\mu _{ATS-ATP}\cong X\left(t\approx 30\,\text{minutes}\right)\cong 0$. After the initial phase, only a more limited random intrafractional motion occurs with spread *σ*_*i*_, which is comparable to the estimate *σ*_*intra* _ obtained in the previous section for the ATS-ATP workflow.

**Table 3:** Fit parameters for drift describing the average start position of the COM at time zero (x_**0**_), the width of the distribution σ_0_ at time zero, the equilibrium intrafractional width of the distribution σ_i_, and the relaxation time τ in minutes for each of the main directions left–right (LR), anterior–posterior (AP), and superior–inferior (SI)

All	*x*_0_[mm]	*σ*_0_[mm]	*σ*_*i*_[mm]	*τ* [min]
LR^a^	0.1	1.1	0.6	12/15
AP	−1.8	3.1	0.8	12.0
SI	1.7	2.6	0.7	14.9

^a^For the LR direction, the data was fitted with the relaxation time fixed to either 12 or 15 min

### Mitigation strategies

The percentage of fractions that required the additional ATP step decreased to 15% when increasing ATP thresholds from 0 to 5 mm. The population mean and systematic error of the motion were greatly reduced using a threshold of 3 mm, and they only slightly decreased with lower threshold values (Table 1).

### Evaluation of 3-mm PTV prostate margins

**Fig. 3:**
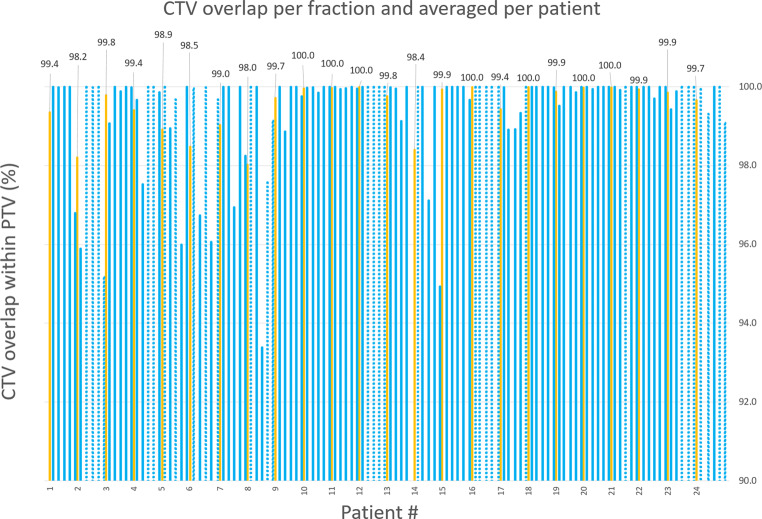
CTV overlap within the PTV as a percentage per fraction (in blue) and averaged per patient (in yellow). Percentage numbers are related to the averaged CTV coverage per patient. Fractions with an additional ATP are marked with dotted bars

After reduction of the prostate PTV margin from 5 to 3 mm, the threshold value for ATP was set to 2 mm, and treatments were closely monitored over the course of treatment. Evaluation of the overlap of the CTV on the “during” MRI with the PTV based on the MRI used for planning was performed for a total of 120 fractions of 24 patients. The overlap range was 93–100% (Fig. 3), and the applied 3‑mm PTV margin for the prostate gland had a 99.5% average geometric CTV overlap for all patients, higher than the 98% threshold we aimed for. An additional ATP adaptation was applied in 33% of all fractions, lower than the 60% we expected from the first 77 patients. Validation of this percentage is not possible, because the number of patients in the dataset is too small. The PTV was reduced by 25% (20 cc) on average by changing the margin from 5 to 3 mm.

## Discussion

This study demonstrated that the ATS-ATP workflow is necessary for an isotropic reduction in the PTV margin from 5 to 3 mm for PCa, mainly to accommodate patients who consistently show a shifting prostate. The motion of the prostate could be described using a random-motion model assuming an out-of-equilibrium initial position of the prostate. A characteristic timescale of 15 min was found, after which only stochastic motion around the prostate’s equilibrium position occurs, with a smaller standard deviation than when fitted on the whole on-couch session. The model explains the success and necessity of the ATS-ATP workflow.

In margin calculations, intrafractional motion is accounted for using a Gaussian distribution with standard deviation *σ*_*i*_, which implicitly assumes that the random motion of the prostate is constrained around an equilibrium position. In other words, the average quadratic displacement is static in time. This contradicts with the observations reported by Ballhausen et al. [11, 30], who indicated that prostate motion resembles an unconstrained random walk, in which the average quadratic displacement increases linearly with time. Additionally, drift motion has been reported [19], implying that the average displacement is linear in time. Our model allows us to understand these different results by taking into account the timescale of the problem. On longer timescales the prostate is constrained and fluctuates around its equilibrium position, whereas on short timescales, a part of the motion is due to drift that leads to larger observed prostate shits. Sampling the motion with higher frequency using cine-MRI [15, 16, 18, 20] could improve the validation of the model and allow for further characterization of the time correlation of the stochastic forces.

The underlying causes of the relaxation process were not investigated but were assumed to be due to the change from the upright to the horizontal position just before the treatment started. Additionally, the filling of the bladder and relaxation of muscles could contribute to this motion.

Improving the speed of the online adaptive workflow, for example by introducing an adapt-to-rotation workflow [26] or by implementing auto-contouring, would require a re-assessment of the possible treatment margins. However, if the time between the ATS and ATP phases is shorter, the prostate will not be in equilibrium, and the gain of the ATS-ATP workflow will not be optimal.

According to the ESTRO-ACROP consensus guideline [31] on the use of image-guided radiation therapy for localized PCa, only intrinsic uncertainties of the image-guided radiotherapy system (0.5–1.5 mm) and intrafractional residual errors remain. This led to a recommendation for PTV margins of between 2 and 4 mm for treatments in five fractions using real-time tracking. This was in agreement with the 3‑mm margin found in this study.

We restricted our analysis of target coverage to the geometric overlap of CTV and PTV. In practice, geometric overlap is more stringent than dosimetric analysis, due to the high conformity and penumbra of the dose. The margin reduction reduced the PTV by 20–50% (mean 20 cc) and resulted in a smaller overlap with the rectum, bladder, and anal canal. This led to a lower dose in the OARs and likely decreased the rates of acute genitourinary and gastrointestinal toxicity [19].

In conclusion, we showed that a PTV margin of 3 mm is clinically possible following an ATS-ATP workflow with a 2-mm ATP threshold, and this is successfully implemented in our clinic.

## Supplementary Information

Supplementary Information shows details of the acquire MR images, used settings for deformable image registration in Plastimatch and detail of the motion model.
