# Correction to: Prostate motion in magnetic resonance imaging-guided radiotherapy and its impact on margins

**DOI:** 10.1007/s00066-025-02390-3

**Published:** 2025-03-13

**Authors:** Johannes Kusters, René Monshouwer, Peter Koopmans, Markus Wendling, Ellen Brunenberg, Linda Kerkmeijer, Erik van der Bijl

**Affiliations:** Department of Radiation Oncology, Radboud university medical center, Nijmegen, The Netherlands

**Correction to:**

**Strahlenther Onkol 2024**

10.1007/s00066-024-02346-z

In this article, the name of the author Johannes Kusters was incorrectly inserted twice in the author line, once as Johannes Kusters and once as Martijn Kusters. Therefore, Martijn Kusters has been removed from the author line.

The correct author line reads as follows: Johannes Kusters^1^ (https://orcid.org/0000-0002-8189-176X) · René Monshouwer^1^ · Peter Koopmans^1^ · Markus Wendling^1^ · Ellen Brunenberg^1^ · Linda Kerkmeijer^1^ · Erik van der Bijl^1^

The original article has been corrected.

