# Supplementary material for: Prostate motion in magnetic resonance imaging-guided radiotherapy and its impact on margins
Source: Strahlenther Onkol. 2025 Jan 14;201(8):799–806. doi: 10.1007/s00066-024-02346-z (PMC12283804; doi:10.1007/s00066-024-02346-z)
Supplement: Supplementary file 1 — Supplementary Information shows details of the acquire MR images, used settings for deformable image registration in Plastimatch and detail of the motion model. [file 66_2024_2346_MOESM1_ESM.docx]

## Supplementary material

#### **Acquired MR imaging sequence**

***MR sequence for pre, pv, during and post MRI scans***Type of acquisition: 3D TSE
Voxel size: 0.83 x 0.83 x 2.0 mm
Field of view: 480 x 480 x 300
Repetition Time (TR): 1535 ms
Echo Time (TE): 277 ms
Bandwidth: 740 Hz/pixel
Flip angle: 90°

***MR sequence parameters for hrMRI***
Type of acquisition: 3D TSE
Voxel size: 0.52 x 0.52 x 0.6 mm
Field of view: 864 x 864 x 417
Repetition Time (TR): 1300 ms
Echo Time (TE): 82 ms
Bandwidth: 753 Hz/pixel
Flip angle: 90°

#### **Details of deformable image registration with Plastimatch**

We developed Python code to perform deformable registration including Plastimatch. A Python wrapper for Plastimatch is used (made by Paolo Zaffino) and was adapted for use with Python 3.8. Deformable image registration (DIR) was performed using Plastimatch (v1.9.3) local registration on the CTV. Plastimach settings for DIR can be found below*.*

The DIR methodology was validated by comparison of the automatically registered prostate COM positions to the clinical ATP after rigid ATS registrations and by calculating the 95% Hausdorff distance (HD95) of deformed contours to clinically delineated contours on pairs of pre MR images. The results of this validation for HD95 were on average 2.9 mm, and the mean surface distance was 1 mm.

The center of mass (COM) position of the CTV prostate on all MR images was calculated from the contours warped by the deformable registration. The COM positions were assigned a time equal to the acquisition timestamps of the MR images.

***Plastimatch settings***

[GLOBAL]

fixed=cropped_moving.nrrd

moving=cropped_fixed.nrrd

xform_out=Bsplinefile.txt

[STAGE]

xform=rigid

optim=versor

impl=itk

metric=mse

max_its=100

convergence_tol=3

grad_tol=1

res=1 1 1

[STAGE]

xform=bspline

optim=lbfgsb

impl=plastimatch

metric=mse

max_its=100

demons_std=10

grid_spac=5 5 5

res=1 1 1

regularization_lambda=0.01

1. **Description and fit of the motion model**

We assumed that equation of motion for each prostate COM x(t) is given by the classic Langevin equation for Brownian motion in a viscous fluid in the presence of a conservative force; for example, see [S1],

$\gamma\dot{x}=-\nabla U+F\left( t \right)$ (S1)

where $\gamma$is the drag-related damping constant and the potential energy is assumed to be of the harmonic form $U\left( x \right)=\frac{{\lambda(x-x_{eq})}^{2}}{2}$, centered around the equilibrium position $x_{eq}$. The last contribution is the stochastic force F(t) which on average is zero, $\left\langle F\left( t \right) \right\rangle=0$, and is uncorrelated in time $\left\langle F(t)F(t') \right\rangle=2D\gamma^{2}\delta\left( t-t' \right)$. The magnitude of the forces is related to the observed diffusion, thus we can introduce the diffusion constant *D* here*.* In the absence of the restoring force resulting from the harmonic potential we obtain the relation for pure Brownian motion $\left\langle{(x\left( t \right)-x(0))}^{2} \right\rangle=6Dt$, which corresponds to the observations by Ballhausen and coworkers [S2].

The resulting Fokker-Planck equation that describes the dynamics of the probability density function $f\left( x,t \right)$ for the Langevin equation (S1) becomes

$$\frac{\partial f\left( x,t \right)}{\partial t}=\frac{1}{\gamma}\nabla\cdot\left( f(x,t)\nabla U(x \right))+D\nabla^{2}(x,t)$$

The quadratic potential energy $U(x)$ yields the equilibrium distribution

$$f_{eq}\left( x \right)\sim e^{-U(x)/\gamma D}$$

This relates the parameters describing the microscopic dynamics within the body to the observed intrafraction standard deviation of the prostate $\sigma_{i}^{2}=\lambda/\gamma D$*.*

At long timescales, the stationary equilibrium distribution is reached. Assuming an initial Gaussian distribution of prostate positions given by $f\left( x,t=0 \right)=N\left( x_{0};\sigma_{0} \right)$.

The solution of the above Fokker-Planck equation subject to this boundary condition yields a generalized Gaussian distribution for the probability density function,

$$f\left( x,t \right)= \frac{1}{\sqrt{2\pi}\sigma\left( t \right)}\exp\left[ -\frac{\left( x-\mu\left( t \right) \right)^{2}}{2\sigma^{2}\left( t \right)} \right]$$

With the time-dependent mean and standard deviation of the distribution given by $\mu\left( t \right)$ and $\sigma\left( t \right)$, which are given in the main text and read

$$\mu\left( t \right)=x_{0}\exp(-t/\tau)$$

$$\sigma\left( t \right)=\sigma_{i}\sqrt{1+\left( \frac{\sigma_{0}^{2}}{\sigma_{i}^{2}}-1 \right)\exp\left[ -2t/\tau\right]}$$

In these equations we introduced the characteristic timescale $\tau$ of the system, which is also related to the microscopic description of the system and given by $\tau=\gamma/\lambda$.

For the averaged position and the squared position we then have

$$\left\langle x(t) \right\rangle= X\left( t \right)$$

$$\left\langle x^{2}\left( t \right) \right\rangle=X^{2}\left( t \right)+\sigma^{2}\left( t \right)$$

The equations for the average and squared average position were fitted simultaneously to the data for the three main directions using the four parameters $x_{0}$, $\sigma_{0}$, $\sigma_{i}$, and $\tau$.

To equate the equilibrium positions $x_{eq}$ for all patients, we defined the patient-specific equilibrium position as the average COM position of the observations taken after 30 min, which is longer than the fitted relaxation time.

[S1] van Kampen NG. Stochastic Processes in Physics and Chemistry. Elsevier; 2007. Chapters 8-9.

[S2] Ballhausen H, Li M, Hegemann NS, Ganswindt U, Belka C. Intra-fraction motion of the prostate is a random walk. Phys Med Biol 2015;60:549-63
